# Cytokines, Adipokines, and Bone Markers at Rest and in Response to Plyometric Exercise in Obese vs Normal Weight Adolescent Females

**DOI:** 10.3389/fendo.2020.531926

**Published:** 2020-12-11

**Authors:** Nigel Kurgan, Katherine McKee, Melissa Calleja, Andrea R. Josse, Panagiota Klentrou

**Affiliations:** ^1^ Department of Kinesiology, Brock University, St. Catharines, ON, Canada; ^2^ Centre for Bone and Muscle Health, Brock University, St. Catharines, ON, Canada; ^3^ School of Kinesiology and Health Science, York University, Toronto, ON, Canada

**Keywords:** exercise, adolescent girls, obesity, inflammation, carboxy-terminal telopeptide, osteocalcin, parathyroid hormone, sclerostin

## Abstract

**Background:**

In adults, excess adiposity has been associated with low-grade, chronic inflammation and compromised bone health, but less is known about these linkages in children. The purpose of this study was to compare the circulating levels of inflammatory cytokines, adipokines, osteokines, and bone markers at rest and in response to plyometric exercise between obese and normal weight adolescent females.

**Methods:**

Ten normal weight (BMI = 21.3 ± 2) and 10 obese (BMI = 32.9 ± 4), postmenarcheal females, aged 13–17 years, performed one bout of plyometric exercise (5 circuits; 120 jumps). Blood samples were taken at rest, 5 min, 1 h, and 24 h post-exercise. Tumor necrosis factor alpha (TNF-α), interleukin 6 (IL-6), insulin, leptin, osteocalcin, carboxy-terminal telopeptide (CTX), sclerostin, and parathyroid hormone (PTH) were measured in serum.

**Results:**

Cytokines were not different between groups at rest or over time with IL-6 increasing (+31%; p = 0.04) 5 min post-exercise and TNF-α decreasing (-9%; p = 0.001) 1 h post-exercise. Insulin and leptin were higher in the obese compared to the normal weight females. In both groups, insulin significantly increased 5 min post-exercise but remained elevated 1 h post-exercise only in the obese group. Leptin did not change in response to exercise. Osteocalcin was lower in the obese group across time and increased (+12%; p = 0.02) 24 h post-exercise in both groups. CTX was similar between groups at rest and decreased (-24%; p < 0.001) 1 h post-exercise. Sclerostin was similar between groups at rest, but there was a significant interaction reflecting a significant increase (+29%; p = 0.04) 5 min post-exercise in the obese group and a non-significant decrease (-13%; p = 0.08) in normal weight controls. PTH increased 5 min post-exercise, dropped 1 h post-exercise to lower than pre-exercise, and returned to baseline 24 h post-exercise in both groups.

**Conclusion:**

Obese adolescent females from our study had no evidence of resting inflammation or differences in bone resorption but show blunted bone formation when compared to normal weight controls. The direction and temporal changes in inflammatory cytokines, adipokines, and bone turnover markers to exercise were similar in both groups, reflecting an overall bone anabolic response for most biomarkers, except sclerostin, which increased only in the obese females immediately post-exercise, suggesting a different systemic regulation of sclerostin depending on adiposity.

## Introduction

Previous studies have suggested that obesity is beneficial to bone due to the increased loading with higher body mass (1). However, there is likely a limit to such benefit, as individuals with over 33% body fat have a negative relationship with bone mineral density (BMD) (2), and overweight adolescent females appear to have lower BMD compared to normal weight females (3, 4). This impairment of the normal growth response of the skeleton (e.g., lower peak bone mass) due to excess adiposity also appears to increase the risk of fracture (5, 6). Thus, understanding the mechanisms that regulate adiposity and bone growth are important as they may aid in mitigating the risk of bone-related diseases and issues, such as osteopenia, osteoporosis, and fractures with increased age.

There are several mechanisms that can explain how obesity may impact bone. Obesity can lead to a state of low grade systemic inflammation, which is associated with increased circulating pro-inflammatory cytokines [e.g. tumor necrosis factor alpha (TNF-α), interleukin 6 (IL-6)] and adipokines (e.g., leptin) and an increase in pro-inflammatory macrophages within adipose tissue (7–9). This inflammation may perpetuate bone loss with aging or attenuate bone growth during adolescence, as higher circulating levels of pro-inflammatory cytokines (10, 11) and adipokines, such as leptin (12, 13), promote bone resorption. Also, obesity promotes the preferential increase in adipocyte formation, which may be at the cost of osteoblastogenesis, since these cells are derived from the same multi-potential mesenchymal stem cells (14). Bone may in turn impact adipose tissue through an endocrine function or molecular crosstalk. For example, *in vivo* ablation of osteocytes leads to a loss of white adipose tissue in murine models (15, 16), suggesting secretory factors from these bone cells (i.e., osteokines) are likely involved in adipose tissue regulation.

Sclerostin is an osteokine that may be implicated in this crosstalk. It is an osteocyte secreted factor that inhibits bone formation by inhibiting Wnt signaling. The Wnt signaling cascade is a critical pathway for the regulation of cell fate and function. Specifically, in bone, Wnt signaling promotes bone formation (17), while in adipose tissue it inhibits adipogenesis (18). Recently, studies have shown that sclerostin promotes adipogenesis *in vitro* (19), and regulates white and brown adipose tissue growth and development in murine models (20–23). In humans, serum sclerostin levels appear to be elevated in individuals with prediabetes and correlate with insulin resistance in skeletal muscle, liver, and adipose tissue (24). In adult females, circulating sclerostin is positively correlated with fat free mass (25). Additionally, systemic low grade inflammation may perpetuate bone loss by inducing the secretion of pro-inflammatory cytokines that are known to stimulate osteoclast activity and subsequent bone resorption (10, 11). Indeed, TNF-α has been shown to increase the expression of sclerostin, which indicates a coordinated control over bone by the induction of inflammation (26, 27). Taken together, these findings suggest a dynamic and coordinated communication between bone and adipose tissue that is likely regulated by inflammation, metabolism, energy demands or adiposity, and may involve the osteokine sclerostin.

Exercise can reduce adiposity and improve bone accrual in adolescence (28–30). Acutely, exercise has also been shown to increase bone formation and decrease bone resorption in both children and adults (31–34). In addition, sclerostin levels have been shown to increase following exercise in adults, while normal weight children show no response (35–38), suggesting children may be protected from sclerostin increasing post-exercise. Cytokine levels can also be altered both acutely and chronically by exercise, resulting in a net-improved, long term, pro-inflammatory profile (39–41). In fact, the acute inflammatory state post-exercise seems to aid, over time, the long-term beneficial muscle adaptations to exercise (42–44). This may be the case for bone and adipose tissue as well. We have previously demonstrated correlations between inflammatory cytokines and bone markers in normal weight young men following a bout of high intensity cycling (34). However, in female adolescents, no data exist examining linkages between the cytokine, adipokine and osteokine response with bone turnover, i.e., the balance between bone formation and bone resorption, either at rest or following exercise, and certainly no data exist examining this relationship in youth with obesity. Thus, the overall purpose of this study was to compare the serum levels of inflammatory cytokines, adipokines, osteokines and bone turnover markers at rest and in response to one bout of plyometric exercise in obese postmenarcheal adolescent females (ObAF) and normal weight postmenarcheal adolescent females (NwAF).

## Materials and Methods 

### Participants

This study represents a secondary analysis that includes data from 20 adolescent females who participated in two larger studies conducted at our institution; half of the NwAF participants were from Dekker et al. (32) with the other half recruited later and all ObAF participants were from Josse et al. (45). Both studies and all procedures received ethical clearance from our University’s Biosciences Research Ethics Board. Further details from both these studies have been published elsewhere (32, 45). For the purpose of the current study, data from 10 NwAF (BMI < 85^th^ percentile, 15.0 ± 1 years of age) and 10 ObAF (BMI ≥ 95^th^ percentile, 14.7 ± 1 years of age), matched for age, menstrual status and somatic maturity, met the inclusion and matching criteria and were selected for the final analysis. Although there were more ObAF participants in the original study (45), we chose 10 of the ObAF participants with the highest BMI percentile who matched appropriately with the 10 available NwAF in all other concordant characteristics to balance the comparison. All participants were postmenarcheal and were tested during the early follicular phase (days 1–7) of their menstrual cycle. In addition, both groups had no previous or current fractures and did not use any pharmaceuticals or nutraceuticals/supplements that may affect bone. Baseline characteristics of the participants are presented in **Table 1**.

**Table 1:** Comparison of anthropometry, body composition and physical activity levels between normal weight and obese girls.

	NwAF (n = 10)	ObAF (n = 10)	p-value
**Age (y)**	15.0 ± 1	14.7 ± 1	0.9
**Age from PHV (y)**	2.6 ± 1	2.5 ± 1	0.8
**Height (cm)**	163.9 ± 7	165.3 ± 7	0.5
**Weight (kg)**	58.6 ± 9	89.9 ± 13	<0.001
**BMI**	21.3 ± 2	32.9 ± 4	<0.001
**Relative body fat (%)**	22.9 ± 5	44.8 ± 4	<0.001
**Waist-to-hip ratio**	0.75 ± 0.04	0.92 ± 0.05	<0.001
**Weekly Physical Activity Score (AU)**	102.4 ± 31	31.9 ± 17	<0.001

Data is presented as mean±SD. PHV, peak height velocity; AU, Arbitrary Units.

### Study Protocol

Both studies followed the same protocol, which involved three sessions in the laboratory. The first session was to inform participants of the study including the risks and benefits, to have them view the laboratory and to obtain informed consent and assent from both the parents/guardians and the participant, respectively. The next two sessions were for testing and took place during the morning hours (between 0800 and 1000 h) to minimize any circadian rhythm variation in the serum biomarkers. For visits two and three, participants were asked to come to the laboratory fasted (~10–12h), and to avoid any vigorous or high-impact exercise for at least 24 h. Upon arrival to the laboratory, if requested, the topical anesthetic cream, Emla (25 mg/g lidocaine + 25 mg/g prilocaine), was applied to the antecubital fossa of the participants arm prior to blood sampling. Subsequently, height, weight and body composition were measured. Participants then sat down and rested for 10 min and had a resting/pre-exercise fasted venous blood sample, which was drawn using a standard venipuncture procedure using a 23G winged infusion set and vacutainers. Following the resting blood sample, participants were provided with a standardized light breakfast (1 granola bar, 1 banana, and water). The breakfast was consistent across both groups and was low in protein, since protein is known to influence markers of bone turnover (46, 47). Within 20 min, they began the 30 min plyometric exercise protocol followed by two more blood samples at 5 min and 1 h post-exercise. Between these two post-exercise blood samples, participants filled out the Godin Shephard Leisure-time physical activity questionnaire, which was used to calculate the weekly leisure activity score (arbitrary units) as previously described (48). Lastly, as participants were postmenarcheal, information regarding menstrual cycles and age of menarche were recorded. Participants returned to the laboratory for the third visit 24 h following the exercise protocol for the last blood sample.

### Exercise Protocol 

The plyometric exercise protocol was designed to provide high-impact, weight-bearing loads. It involved 120 jumps organized into five circuit training stations (three sets of eight repetitions, with 3 min of recovery between sets) (49). The five circuit stations included box, lunge, and tuck jumps, single leg hopping, and jumping jacks. The same protocol has been previously used in our laboratory and was successful in eliciting responses in bone biomarkers in pediatric populations (31, 32, 35, 38). 

Participants began the exercise session with a warm-up that included 5 min of low-intensity cycling (~40W of resistance) on a cycle ergometer. Once the warm-up was completed, participants were given a comprehensive explanation and demonstration of each of the circuits. They were also allowed to familiarize themselves with each circuit to ensure proper technique and form to minimize injury. Jump height for box jumps was set at 40 cm for the NwAF and 25 cm for the ObAF. The height difference between the boxes was meant to control for differences in ground reaction forces between the two groups due to the obese girls having significantly higher body mass. Though the precise load required to elicit an osteogenic response in youth is still unknown, the jump height was adjusted for the adolescent females to reflect differences in weight (e.g., have comparable ground reaction forces) and to ensure safety. Each plyometric testing protocol was carried out by 2 research/study staff to ensure safety, and form/technique was watched closely to minimize any risk of injury. There were no adverse events because of the exercise protocol in this study.

### Measurements

Height and seated height were measured with a stadiometer (Ellard Instruments, Monroe WA, USA) to the nearest 0.1 cm with no shoes and light clothes. These measures were then used to assess somatic maturity, expressed as years from age of peak height velocity (aPHV) as previously described (50). Body mass (kg) and body composition, including lean body mass (LBM), fat mass (FM), and relative body fat percent (%BF), were measured using bioelectrical impedance analysis (BIA; InBody520 bioelectrical impedance analysis system; Biospace Co. Inc. Los Angeles, CA, USA).

Venous blood was collected into serum separator vacutainers (cat#: 367983-1, BD, Mississauga, ON) and clotted for ~15 min before being centrifuged at ≤1400 RCF (g) for 15 min. The serum was separated and aliquoted into polyethylene tubes for storage at –80°C until analysis upon study completion. Immediately following each blood sample, hematocrit was measured to test for potential post-exercise hemoconcentration (shifts in plasma volume) using the microhematocrit method (51). Relative change in plasma volume (%ΔPV) from pre- to post-exercise was estimated using the Van Beaumont formula (51). No changes in plasma volume between pre- and post-exercise were observed, therefore unadjusted concentrations were presented. 

### Blood Biomarkers

All samples used for the purpose of this secondary analysis were reanalyzed together. Serum sclerostin was analyzed in duplicate using an enzyme linked Immunosorbent assay (ELISA; cat# DSST00; R&D, Minneapolis, MN). The average intra-assay coefficient of variation for sclerostin was 3.8%, and the inter-assay coefficient of variation was 5.1%. Osteocalcin, parathyroid hormone (PTH), insulin, leptin, IL-6 and TNF-α were analyzed in duplicate using Milliplex MAP human bone magnetic bead panels (HBNMAG-51K; EMD, Millipore Corporation, Etobicoke, ON). The average intra-assay coefficient of variations for IL-6, TNF-α, osteocalcin, PTH, insulin and leptin were 9.5, 5.0, 5.4, 7.9, 6.5, and 6.5%, respectively. The average inter-assay coefficient of variation for all analytes was 6.9%. β−isomerized Carboxy−terminal cross-linking telopeptides (CTX) (cat#: 11972308 122, β-CrossLaps) was measured from serum at the Mount Sinai Hospital Core Laboratory (Toronto, Ontario) using a Roche Cobas e602 Modular Analytics automated analyzer. Lower and upper detection limits were 0.010–6.00 ng/ml (quality control standard CV: 4.8%).

### Statistical Analysis

Normality was confirmed using the Kolmogorov-Smirnov test, z-scores for skewness and kurtosis and visual screening of box plots. Unpaired/independent t-tests were used to assess physical characteristics and maturity between groups. A series of two-way repeated measures ANOVAs were used to assess time and group effects and time-by-group interactions for each biomarker and confidence intervals were adjusted based on Least Significant Difference (LSD), as all biomarkers did not pass Mauchly’s Test of Sphericity. In the event of a significant time-by-group interaction, further pairwise comparisons were made using one-way repeated measures ANOVAs for each group with Tukey’s multiple comparison test. Significance was accepted at an alpha level of <0.05 for all analyses. Statistical Analysis was performed using SPSS version 25 for Windows.

## Results

As per the study design, there were no significant differences in age, height and somatic maturity between NwAF and ObAF, while ObAF had significantly higher body mass, BMI, %BF and waist-to-hip ratio, and lower weekly leisure activity score than NwAF (**Table 1**).

**Figure 1:**
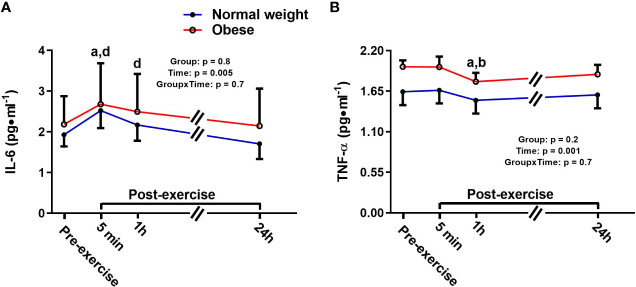
Serum concentrations (mean±SEM) of interleukin 6 (IL-6, **A**) and tumor necrosis factor alpha (TNF-α, **B**) pre- and post-exercise in normal weight (NwAF) and obese adolescent (ObAF) females. For post-hoc analysis for combined groups (black letters): a = significant difference from pre-exercise (p ≤ 0.05); b = significant difference from 5 min post-exercise (p ≤ 0.05); d = significant difference from 24 h post-exercise (p ≤ 0.05).

There was a significant main effect for time for IL-6, with no main effect for group or time-by-group interaction. Specifically, IL-6 increased 5 min following the plyometric protocol compared to pre-exercise [+0.6 pg/ml (+31%), p = 0.02], then returned to baseline 24 h post-exercise in both groups (**Figure 1A**). TNF-α also showed a significant main effect for time, but no significant main effect for group or time-by-group interaction. In both groups, TNF-α was significantly lower at 1h post-exercise compared to both pre-exercise [-0.16 pg/ml (-9%), p = 0.001] and 5 min post-exercise [-0.15 pg/ml (-8%), p = 0.001] concentrations (**Figure 1B**).

**Figure 2:**
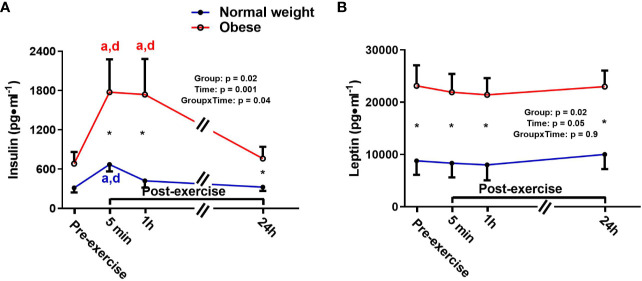
Serum concentrations (mean±SEM) of insulin **(A)** and leptin **(B)** pre- and post-exercise in normal weight (NwAF) and obese adolescent (ObAF) females. For post-hoc analysis within normal weight females (blue letters) and obese females (red letters): a = significant difference from pre-exercise (p ≤ 0.05); b = significant difference from 5 min post-exercise (p ≤ 0.05); c = significant difference from 1 h post-exercise (p ≤ 0.05); d = significant difference from 24 h post-exercise (p ≤ 0.05). *Significant mean difference between groups (p ≤ 0.05).

There were significant main effects for time and group, as well as a significant time-by-group interaction for insulin, indicating that the ObAF had higher insulin levels than NwAF across all tome points [mean difference: 787 pg/ml (174%), p = 0.03] and responded differently over time to exercise and feeding (**Figure 2A**). Insulin significantly increased from pre- to 5 min post-exercise [+345 pg/ml (+108%), p = 0.001] and returned to baseline 1h post-exercise in NwAF (**Figure 2A**). The ObAF also increased from pre- to post-exercise [+1,091 pg/ml (+129%), p = 0.02], but their levels remained elevated 1 h post-exercise and returned to baseline 24 h post-exercise, indicating the exercise and feeding-induced increase in insulin was higher and more prolonged in ObAF compared to NwAF (**Figure 2A**). There was a significant main effect for group for leptin, with the ObAF having significantly higher leptin levels than NwAF at all time points [mean difference: 13,579 pg/ml (+2.6-fold), p = 0.02] with no significant time effect and no significant interaction (**Figure 2B**).

**Figure 3:**
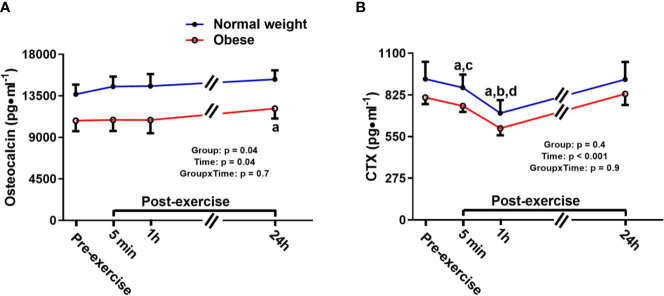
Serum concentrations (mean±SEM) of osteocalcin and CTX pre- and post-exercise in normal weight (NwAF) and obese adolescent (ObAF) females. For post-hoc analysis for combined groups (black letters): **(A)** significant difference from pre-exercise (p < 0.05); **(B)** significant difference from 5 min post-exercise (p ≤ 0.05); c = significant difference from 1 h post-exercise (p ≤ 0.05); d = significant difference from 24 h post-exercise (p ≤ 0.05). *Significant mean difference between groups (p ≤ 0.05).

Osteocalcin showed a significant main effect for group and time, but no time-by-group interaction. The main time effect reflected a similar exercise induced increase in bone formation/turnover following plyometric exercise in both groups, and both groups had a progressive increase in osteocalcin after exercise, reaching a significant difference 24 h post-exercise compared to pre-exercise [+1,464.6 pg/ml (+12%), p = 0.04]. The main group effect demonstrated that the ObAF had significantly lower osteocalcin levels compared to NwAF [mean difference: 3,326 pg/ml (-23%), p = 0.04] (**Figure 3A**). CTX showed a significant main effect for time, but no group effect or time-by-group interaction, suggesting similar exercise-induced bone resorption changes between ObAF and NwAF. Compared to pre-exercise, CTX significantly decreased 5 min [-156 pg/ml (-19%), p < 0.001] and 1 h following the plyometric exercise protocol [-204 pg/ml (-24%), p < 0.001], then returned to baseline 24 h post-exercise in both groups (**Figure 3B**).

**Figure 4:**
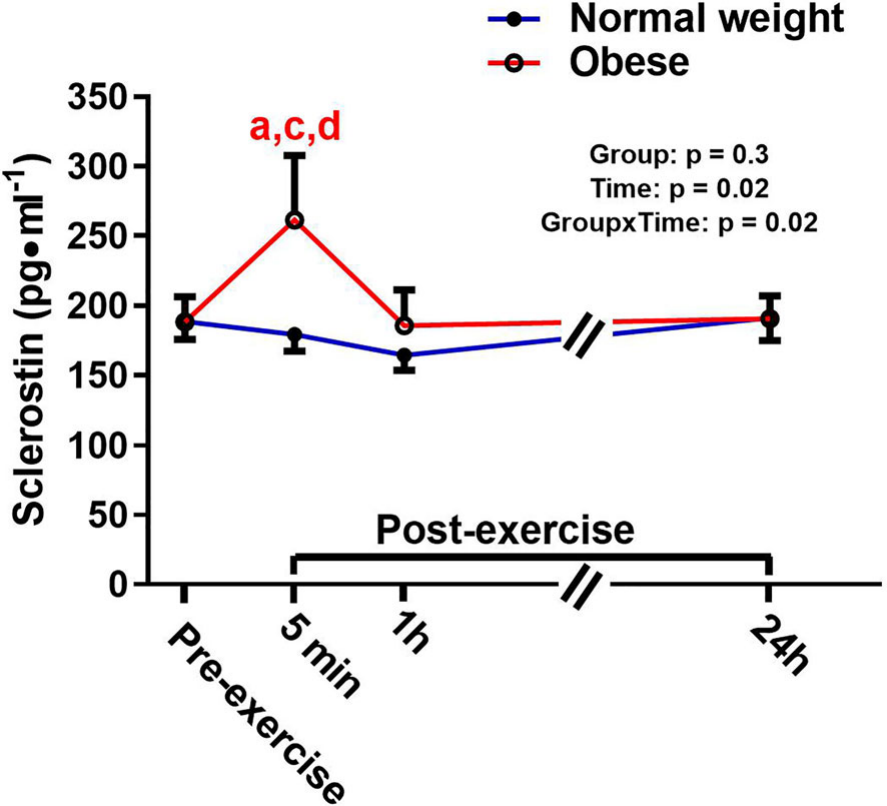
Serum concentrations (mean±SEM) of sclerostin pre- and post-exercise in normal weight (NwAF) and obese adolescent (ObAF) females. For post-hoc analysis within normal weight females (blue letters) and obese females (red letters): a = significant difference from pre-exercise (p ≤ 0.05); c = significant difference from 1 h post-exercise (p ≤ 0.05); d = significant difference from 24 h post-exercise (p ≤ 0.05).

**Figure 5:**
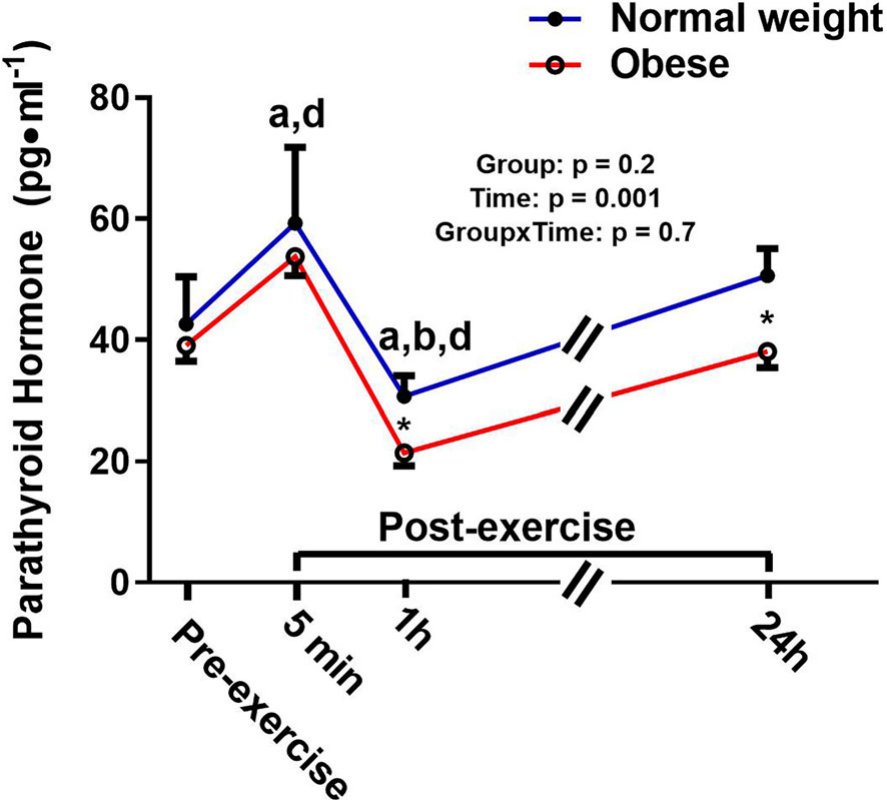
Serum concentrations (mean±SEM) of parathyroid hormone (PT) pre- and post-exercise in normal weight (NwAF) and obese adolescent (ObAF) females. For post-hoc analysis for combined groups (black letters): a = significant difference from pre-exercise (p ≤ 0.05); b = significant difference from 5 min post-exercise (p ≤ 0.05); d = significant difference from 24 h post-exercise (p ≤ 0.05). *Significant mean difference between groups (p ≤ 0.05).

There was a significant main effect for time and a significant time-by-group interaction for sclerostin, but no significant group effect (**Figure 4**). The interaction reflected that the groups responded differently to the exercise bout. Upon further inspection, this differential response was mostly driven by the ObAF that had a significant increase in sclerostin from pre- to 5 min post-exercise [+73 pg/ml (+26%), p = 0.04] that returned to baseline 1 h post-exercise, while the NwAF had no change in sclerostin immediately post-exercise (**Figure 4**). PTH showed a significant main effect for time, but no group effect or time-by-group interaction (**Figure 5**). Specifically, PTH levels significantly increased 5 min post-exercise compared to pre-exercise [+15.7 pg/ml (+38%), p = 0.05], dropped 1 h post-exercise to lower than pre-exercise [-14.8 pg/ml (-36%), p = 0.001], and returned to baseline 24 h post-exercise in both groups combined (**Figure 5**).

Percent changes are presented in **Supplementary Table 1** for all serum biomarker concentrations at 5 min, 1 h, and 24 h post-exercise relative to pre-exercise values within each group.

## Discussion

This is the first study to compare the resting levels of serum osteokines, inflammatory cytokines, and adipokines and their responses to a single bout of high-impact, plyometric exercise in postmenarcheal adolescent females with normal weight (NwAF) and obesity (ObAF). There were three main findings from this study. First, there was no evidence of chronic low-grade inflammation in these ObAF. In addition, the similar cytokine response to exercise between groups suggests an exercise induced anti-inflammatory response that is intact even in the presence of excess adiposity. Second, compared to the NwAF, the ObAF had a significantly lower concentration of osteocalcin, both at rest and in response to acute exercise, suggesting that obesity during adolescence may blunt bone turnover, and thus prevent normal bone growth over time. Despite a global mean difference in osteocalcin levels and no difference in CTX, the response to plyometric exercise in these markers were similar between NwAF and ObAF, as both showed a transitory decrease in bone resorption (i.e., CTX) 1 h post-exercise and an increase in bone formation/turnover (i.e., osteocalcin) 24 h following plyometric exercise. Third, there was a differential response of sclerostin to exercise both directionally and temporally, as ObAF had an increase 5 min post-exercise while NwAF had no significant response.

### Inflammatory Cytokines at Rest and in Response to Plyometric Exercise

There was no difference in the resting levels of IL-6 or TNF-α between NwAF and ObAF. These findings are in contrast to previous studies that have reported small, yet statistically significant, elevations in resting IL-6 and TNF-α levels in inactive 10–13 year old children with obesity compared to normal weight controls (52, 53). In contrast, when children with obesity were as active as children with healthy body fat levels, there were no differences in systemic inflammation (53). Our findings do not support this protective mechanism of inflammation with higher physical activity levels, since the ObAF reported significantly lower leisure time activity than the NwAF despite having similar cytokine profiles. In contrast, the ObAF may have surpassed a “threshold” of weekly physical activity that was sufficient to protect them despite their levels being significantly lower than the NwAF, as there levels appear to be similar to normal weight adults (54). Alternatively, our results may simply indicate that there was no detectable systemic low-grade inflammation in this cohort of ObAF independent of physical activity/exercise training. This is in line with the idea that overt systemic inflammation is not always detectable in children, but that an early, potentially local (e.g., in adipose depots), induction of inflammation in children with obesity may precede detectable levels within the circulation that are typically observed in adults with obesity (41, 55). The accumulation of (visceral) adipose tissue from childhood to young adulthood likely precedes the induction of chronic low-grade systemic inflammation seen more overtly in adult populations (56). Thus, our findings reinforce that adolescence is a critical time for intervening to reduce adiposity in hopes of preventing systemic inflammation and associated chronic diseases later in life (41, 44, 57).

There were no differences in the cytokine response to the acute plyometric exercise bout between ObAF and NwAF. Despite the significant differences in body mass and fat between the groups there was an identical transient response in IL-6 in direction and magnitude, which indicates that this type of exercise was sufficient at eliciting an IL-6 response to exercise in both groups. Although this has not been confirmed, the IL-6 increase post-exercise was likely secreted by the muscle and driven by changes in calcium, reactive oxygen species and/or substrate utilization [e.g., glycogen (58)] (56) in response to exercise. This implies that these mechanisms resulting in the release of IL-6 are still intact in adolescents with obesity. In contrast, the TNF-α response to plyometric exercise in our adolescent females was not similar to what is typically observed in adults. Specifically, TNF-α has been shown to increase significantly or remain unchanged post-exercise in adults (44, 59, 60). Here we report that TNF-α significantly decreased 1h post-exercise. While mechanistically this may make sense, given the increase in IL-6 is known to increase anti-inflammatory cytokines (e.g., IL-10) and reduce TNF-α production (39, 44, 55, 61), this small decrease (-5 and -9% from pre- to 1 h post exercise in NwAF and ObAF, respectively) may not be clinically relevant.

### Leptin and Insulin at Rest and in Response to Plyometric Exercise

Leptin is an adipokine that acts to stimulate appetite and diminishes energy expenditure to maintain fat stores, thus playing a role in energy homeostasis (62). In this study, leptin; was significantly higher at all time points in the ObAF (consistent with their greater fat mass) compared to NwAF and unaffected by an acute bout of exercise in both groups. Exercise training induced reductions in fat mass are normally needed to see reductions in circulating leptin (63, 64). Moreover, elevated leptin levels with obesity are likely also a result of leptin resistance, causing its overproduction (65). Increased leptin levels are associated with insulin resistance, which, in turn, can lead to hyperinsulinemia (66). Indeed, we did find evidence of hyperinsulinemia post-exercise only in the ObAF (after consuming a high carbohydrate breakfast and exercising), which remained elevated for longer compared to NwAF. This indicates a potential impairment in postprandial glucose metabolism/handling in the ObAF. Leptin also has immune modulating effects (67), which include inducing the production of pro-inflammatory cytokine (e.g., TNF-α and IL-6), reactive oxygen species, and lymphopoiesis (65, 68–73). While there were no differences in circulating inflammatory cytokines between groups in this study, chronically elevated leptin may induce systemic inflammation that will be detectable over time (74). Thus, elevated leptin in youth may trigger local/tissue level inflammation that has not yet manifested as systemic inflammation, but still has adverse consequences.

### Bone Turnover and Sclerostin at Rest and in Response to Exercise

NwAF had a significantly higher concentration of osteocalcin compared to ObAF at rest, suggesting that obesity during adolescence may blunt bone turnover and prevent normal bone growth over time (75). Despite this difference at baseline, the direction and magnitude of the response of these markers to plyometric exercise was similar between NwAF and ObAF, as both showed a transitory decrease in bone resorption (i.e., CTX) 1 h post-exercise and an increase in bone turnover/formation (i.e., osteocalcin) 24 h following high impact exercise. These findings are in line with previous studies that highlight a similar osteogenic response to acute exercise (31, 34) in male adults and children and a lower rate of bone turnover, and particularly lower bone formation, at rest in adolescents and young adults with obesity (5, 76). Taken together, it appears obesity in adolescence decreases bone turnover at rest, which may explain why children with obesity tend to present with an earlier achievement, and lower, peak height velocity, ultimately resulting in impaired linear growth (77). It is important to point out that our participants were matched for somatic maturity; hence, there was no significant difference in the age from peak height velocity between groups. Alternatively, the lower rate of bone turnover may affect bone quality. Studies have demonstrated that while adolescents with obesity tend to have increased bone mass, they also have an increased risk of fracture (78, 79) and a relatively lower bone mass for their weight (80). While there were differences in bone turnover at rest, the osteogenic bone turnover (i.e., CTX and OC) response to high-impact plyometric exercise was similar between groups. This suggests that higher impact loading exercise is beneficial for promoting positive bone turnover responses during this critical period for bone accrual (i.e. adolescence) (81) independent of adiposity or body mass.

There was a differential response in sclerostin post-exercise in the NwAF and ObAF. Specifically, NwAF had no change in sclerostin, while the ObAF showed a significant increase 5 min post-exercise which returned to baseline 1h post-exercise. The response in the NwAF was consistent with what we have previously reported in pre- and postmenarcheal females (32). Interestingly, the ObAF response was similar to what we have previously observed in adult females of normal weight (37). This finding supports previous reports that obesity in adolescence may promote advanced linear growth and skeletal maturation (i.e., “bone aging”) (82), which may have a negative impact on normal linear bone growth and development (77). Although we are unclear of what may be driving this differential response, the fact that adolescents with obesity have similar bone responses to adults following impact exercise warrants further investigation.

Research suggests that PTH and TNF-α may play a role in regulating sclerostin’s response to exercise since they have been shown to decrease (83, 84) and increase (27, 59) sclerostin expression, respectively. While both PTH and TNF-α fluctuated with exercise in this study, their responses were similar between the two groups, suggesting that PTH and/or TNF-α cannot explain the differential response of sclerostin post-exercise between our two groups. Alternatively, the exercise response in the ObAF may merely reflect higher sclerostin content within the bone microenvironment (e.g., canalicular-lacunar network) (35) that is released/secreted during mechanical loading (i.e., acute exercise). We initially hypothesized that there may be tissue crosstalk from adipose tissue to bone, in the form of inflammation, that would result in increasing sclerostin levels, both at rest and in response to exercise. This would be more apparent in the ObAF compared to NwAF. While we did not observe any differences in inflammation between ObAF and NwAF, there is an influence of obesity on the regulation of sclerostin post-exercise. This finding is intriguing given that humans who have mutations that inhibit Wnt signaling (i.e., sclerostin’s mechanism of action) show increased bone loss and alterations in the regulation of fat mass and energy balance (85, 86). Moreover, murine models highlight an important role of sclerostin in regulating adipose tissue metabolism and growth and development (20–23). This further points towards sclerostin as being an endocrine factor that likely plays a role in regulating tissues beyond bone (e.g., adipose tissue).

This study adds to a body of literature examining the effect of obesity on factors that regulate inflammation, bone turnover, and adiposity in adolescent females, which is an understudied population and a critical period of growth and development. While there were several novel findings in this study, there were also some limitations; first, the small sample size may have contributed to the lack of detection of significant differences post-exercise, particularly in inflammatory cytokines. Second, we did not measure all bone-related biomarkers, including, for example, blood calcium levels, which could have provided some insight regarding the fluctuations we observed in serum PTH. In addition, participants ate a small standardized breakfast before performing the exercise bout, which is known to impact some of the serum biomarkers we measured [e.g., IL-6, insulin, and bone turnover markers (CTX)]. There were also differences between groups in other variables such as habitual physical activity levels in addition to body mass and body composition (by design). While we believe that differences in adiposity are most responsible for driving these differences, it is unclear whether other differences between groups, such as habitual physical activity levels, may also have played a role. Lastly, the exercise mode used in this study may have provided small differences in mechanical loading between the groups. This was a result of the ObAF having a larger body mass and the NwAF having a higher vertical jump. While we tried to reduce the height of the box jumps for the ObAF compared to the NwAF to account for this, there still may have been small differences. Despite this potential difference, both groups responded similarly to the plyometric protocol, therefore we can assume both groups had a sufficient loading stimulus. Examination of the influence of longer-term exercise and dietary interventions (i.e. lifestyle modification) on the acute response of cytokines, osteokines and adipokines in pediatric populations should be done to see if these differences persist/change over time with interventions designed to reduce adiposity and improve fitness and overall health. Mechanistic studies should also be done (in animal models) to examine the importance of sclerostin to the exercise induced adaptations of adipose tissue.

## Conclusion

This study found no detectable differences in inflammatory cytokines at rest or post-exercise between the adolescent females with normal weight and obesity. This study also provided evidence that a single bout of plyometric exercise can lead to an acute anti-inflammatory response post-exercise, as both ObAF and NwAF showed an increase in IL-6 at 5 min post-exercise and a decrease in TNF-α 1h post-exercise. There was also a steady decrease in CTX 1h post-exercise and a similar increase in osteocalcin 24 h post-exercise in both groups of adolescent females, suggesting an overall anabolic bone response to loading exercise in this population. Additionally, in the fasted rested state, osteocalcin was significantly lower in participants with obesity, indicating an overall lowering of bone turnover, which overtime may impact normal bone growth and development. Finally, we observed no difference in resting sclerostin levels, but there was a differential response post-exercise where the ObAF had a significant increase in sclerostin immediately post-exercise while the NwAF had no response. The impact of this differential regulation post-exercise is not known but is a cause for concern given sclerostin’s role in positively regulating adipogenesis and negatively regulating osteogenesis.

## Data Availability Statement

The datasets generated for this study are available on request to the corresponding author.

## Ethics Statement

The studies involving human participants were reviewed and approved by Brock University Research Ethics Board. Written informed consent to participate in this study was provided by the participants’ legal guardian/next of kin.

## Author Contributions

NK contributed to data collection, performed the analysis of blood samples, completed the statistical analysis and prepared the first draft of the paper. KM and MC contributed to data collection and analysis. AJ contributed to the experimental design and the interpretation of the data. PK was the principal investigator of the research and contributed to the experimental design, data analysis and interpretation of the data. She is the corresponding author. All authors contributed to the article and approved the submitted version.

## Funding

This study was funded by a Natural Sciences and Engineering Research Council of Canada (NSERC) grant to PK (grant # 2015-04424). This study was also partially funded by a Dairy Farmers of Canada grant to AJ and PK. NK holds an NSERC doctoral scholarship. Support for the open access publication fees was received through the Brock University Library Open Access Publishing Fund.

## Conflict of Interest

The authors declare that the research was conducted in the absence of any commercial or financial relationships that could be construed as a potential conflict of interest.
