# Supplementary material for: Cytokines, Adipokines, and Bone Markers at Rest and in Response to Plyometric Exercise in Obese vs Normal Weight Adolescent Females
Source: Front Endocrinol (Lausanne). 2020 Dec 11;11:531926. doi: 10.3389/fendo.2020.531926 (PMC7759614; doi:10.3389/fendo.2020.531926)
Supplement: Supplementary file 1 [file Table_1.docx]

**Supplementary Table 1.** Percent changes in serum biomarkers from baseline to 5 min, 1h and 24h following plyometric exercise in normal-weight adolescent females (NwAF) and obese adolescent females (ObAF).

|  | **5 min Post-Exercise**  **(%Δ from Pre-Exercise)** | | **1h Post-Exercise**  **(%Δ from Pre-Exercise)** | | **24 h Post-Exercise**  **(%Δ from Pre-Exercise)** | |
| --- | --- | --- | --- | --- | --- | --- |
|  | **NwAF** | **ObAF** | **NwAF** | **ObAF** | **NwAF** | **ObAF** |
| **IL-6** | 60±41 | 27±15 | 2.3±7.4 | 8.2±8.5 | -5.4±11.9 | -7.5±10.4 |
| **TNF-α** | 2±3 | 2±3 | -5.0±2.7 | -8.6±2.4 | -3.4±2.0 | -3.4±4.6 |
| **Insulin** | 108±24 | 129±55 | 26.1±9.5 | 92.6±27.3***** | 10.4±12.2 | 16.1±11.9 |
| **Leptin** | -5±4 | -1±4 | -17.5±3.3 | -2.6±4.6***** | 20.8±15.9 | 4.3±3.9 |
| **Osteocalcin** | 7±3 | 6±6 | 3.1±3.8 | 6.1±7.0 | 20.1±12.2 | 16.5±7.1 |
| **CTX** | -5±4 | -7±3 | -23.9±6.7 | -25.8±2.5 | -0.4±3.3 | 1.5±5.8 |
| **Sclerostin** | -2±3 | 26±12***** | -6.5±5.1 | -3.7±5.1 | 3.1±6.3 | 2.7±5.0 |
| **Parathyroid Hormone** | 35±20 | 41±9 | -25.4±9.3 | -42.7±7.2 | 1.7±8.8 | 2.1±10.8 |

*****denotes significant difference (p<0.05) between groups in post-hoc pairwise comparisons; IL-6=interleukin 6; TNF-α=tumor necrosis factor alpha; CTX=carboxy-terminal telopeptide; Data presented as Mean±SEM.
